# A critical review of the Online Safety Bill

**DOI:** 10.1016/j.patter.2022.100544

**Published:** 2022-06-24

**Authors:** Markus Trengove, Emre Kazim, Denise Almeida, Airlie Hilliard, Sara Zannone, Elizabeth Lomas

**Affiliations:** 1 School of Public Policy, University College London, 29 Tavistock Square, London WC1H 9QU, UK; 2 Holistic AI, 18 Soho Square, London W1D 3QL, UK; 3 Department of Computer Science, University College London, Gower Street, London WC1E 6EA, UK; 4 Department of Information Studies, University College London, Gower Street, London WC1E 6BT, UK; 5 Institute of Management Studies, Goldsmiths, University of London, New Cross, London SE14 6NW, UK

**Keywords:** online safety bill, digital ethics, online harms bill, privacy, free speech, artificial intelligence

## Abstract

The UK Parliament has tabled the Online Safety Bill to make the internet safer for users by requiring providers to regulate legal but harmful content on their platform. This paper critically assesses the draft legislation, surveying its rationale; its scope in terms of lawful and unlawful harms it intends to regulate; and the mechanisms through which it will be enforced. We argue that it requires further refinement if it is to protect free speech and innovation in the digital sphere. We propose four conclusions: further evidence is required to substantiate the necessity and proportionality of the Bill’s interventions; the Bill risks a democratic deficit by limiting the opportunity for parliamentary scrutiny; the duties of the bill may be too wide (in terms of burdening providers); and that enforcement of a Code of Practice will likely be insufficient.

## The bigger picture

This critical perspective makes a timely contribution to the tech policy debate concerning the monitoring and moderation of online content. Governments globally are currently considering a range of legislative interventions to limit online abuse, disinformation, and the dissemination of illegal content on social media platforms. These interventions will significantly impact online free speech, competition between platforms, and the democratic function of online platforms. By investigating the UK’s Online Safety Bill, comparing it with similar interventions, and considering the political impact of different digital tools for moderation, this perspective aims to inform the current policy debate by combining technical and political insight. It indicates the need for further research into the comparative efficacy of different methods of content monitoring and moderation.

## Teaser

Governments, including the UK, the EU, Brazil, and India, are legislating to moderate harmful online speech. This perspective compares approaches to this problem, focusing on the UK’s Online Safety Bill, to highlight the challenges governments face in balancing online safety, free speech, and competition in the digital economy. It argues that the UK’s intervention is problematic for free speech and democracy, and so we argue for an ethics-by-design approach to moderation instead.

## Introduction: Online regulation in the UK

The internet has become increasingly integrted into our individual and communal lives: over 90% of UK citizens are now online. Indeed, with this, existing social maladies have found a new, and complex, expression: child abuse, terrorist propaganda, and the harassment of women and minority groups are among the phenomena that have been transformed by the internet, and that have rendered it an unsafe place for many of its netizens.^1^ To cite but a few statistics to this effect: the Internet Watch Foundation confirmed 153,383 cases of Child Sexual Abuse Material (CSAM) in the UK in 2020^2^; the UK government claimed that all five domestic terrorist incidents in 2017 had online elements, including radicalization by international groups, such as ISIS^3^; 21% of women in the UK have been victim to misogynistic abuse online^4^; two in every three Britons are concerned about the proliferation of fake news.^5^

The internet seems a conducive environment for these harms: it is a space that sprawls jurisdictions, it develops at a faster pace than regulation, and it allows great degrees of anonymity and secrecy for those wanting to commit wrongdoing with impunity. Despite reporting concerns about their safety and the paucity of protective measures,^6^ Britons still enter online spaces—largely because of the benefits of internet services, but also out of a perceived lack of plausible alternatives, and because of an increasing truism that the internet is effectively an extension of the public square.

It is against this background that the British Parliament is now considering the Online Safety Bill (previously drafted with the title of “The Online Harms Bill”).^7^ The purpose of the bill is to create “a new regulatory regime to address illegal and harmful content online.”^7^ Key among the stipulated objectives of the legislation are the following:

• *A free, open and secure internet*:^1^ individuals must be able to use the internet without restriction, except where limited and proportionate restriction is necessary for protecting individuals’ rights and interests.
• *Freedom of expression online*: each person has the right to express themselves freely and to receive information from others, unless where such expression is prohibited by law, as in the case of hate speech or terrorist propaganda. This right includes the expression and transmission of information online.
• *An online environment where companies take effective steps to keep their users safe, and where criminal, terrorist, and hostile foreign state activity is not left to contaminate the online space*:^1^ recent controversies—including allegations of Russian social media interference in elections, and terrorist recruitment online by ISIS—have emphasized the need to protect users on social media sites from bad-faith actors.
• *Rules and norms for the internet that discourage harmful behavior*:^1^ features of user-to-user services (including the possibility of anonymity) seem to encourage antisocial behavior.
• *The UK as a thriving digital economy, with a prosperous ecosystem of companies developing innovation in online safety*:^1^ user-to-user and search services are sites of innovation and growth. Accordingly, they are of great importance to the development of the digital economy. The UK has also been party to world-leading safety innovation previously, in the form of General Data Protection Regulation (GDPR).
• *Citizens who understand the risks of online activity, challenge unacceptable behaviors and know how to access help if they experience harm online, with children receiving extra protection*:^1^ user-to-user services potentially expose vulnerable people—particularly children—to bad-faith actors. It is therefore important to ensure that there is sufficient protection to make their internet use secure.
• *Renewed public confidence and trust in online companies and service*s:^1^ controversies involving user-to-user and search services have undermined public trust in their reliability as news sources and service providers.

Given the extent of such harms, such policy objectives are commendable; in particular, because they are cognisant of—and indeed affirm—the need for a safe, free, open, and thriving digital environment. In this paper, we critically analyze the mechanisms and rationales for the regulation that the British government has proffered, arguing that the proposed legislation fails to meet the government’s own desiderata. The effect of this legislation, in our opinion, will be a less open and free internet, one in which British companies have less access to a thriving digital economy, and in which the most effective steps toward preventing harm are neglected. Per our analysis, the government has not done enough to justify the need for its intervention, and has crafted a regulatory framework that leaves open the possibility of counterproductive and undemocratic interference.

To motivate our opinion, we offer a selected overview of the proposed legislation, where our selection of emphasis is presented with a view to offering commentary on those specific elements. Recognizing that this space is of critical public concern and presents genuinely novel forms of policy interaction (from duty of care; protection of the vulnerable; digital and algorithmic justice; privacy; respect, dignity, and decency in society; freedom of expression; etc.), readers will notice that our interventions highlight points of contestation, which, at a high level, represent calls for further evidence and greater consultation with relevant stakeholders.

We forward our critical review with two main sections: the first is an overview, where we summarize the key components of the Bill and its preceding White Papers.^1^ Here, we survey the following:

• *Rationale*: drawing on the government’s White Paper, we reconstruct their explanation for the necessity of the Bill, including the social problems it aims to correct and the regulatory lacuna it aims to fill.
• *Scope*: we survey the wide range of unlawful and lawful harms on digital services that the Bill intends to regulate.
• *Enforcement*: we survey the mechanisms through which the Bill enforces regulation, particularly by empowering the regulator, Ofcom, and the Minister.
• *International comparisons*: we survey similar regulatory proposals from other jurisdictions aimed at resolving the same set of issues.

Secondly, we offer our critical commentary on these features of the Bill. In our critical discussion, our main conclusions are the following:

• *Further evidence needed*: we argue that the government’s White Papers for the Online Harms Bill and Online Safety Bill do not provide sufficient evidence for the necessity or efficacy of regulatory intervention. Despite the prevalence of harms online, it is not clear from the White Papers why extensive government interference—with its concomitant limitations on freedom and individual rights—is a necessary or proportionate resort in resolving these issues.
• *Possible democratic deficit*: we argue that the Online Safety Bill suffers a possible democratic deficit because it delegates extensive authority to Ofcom in its capacity as the industry regulator for digital media, and to the Minister. In assigning Ofcom the power to determine the Code of Practice for digital platforms, the Online Safety Bill empowers Ofcom with sweeping powers to enact rules for the internet with little democratic scrutiny by Parliament and no consultation.
• *Duties too wide*: we are concerned about the potential range of new powers and duties created by the Online Safety Bill. The Online Safety Bill extends services’ duty of care to include the regulation of legal but harmful material. We argue both that this extension overburdens developers with responsibility—at pain of penalty—for legal content and that the specific framing of this provision risks a regulatory slippery slope toward wider censorship.
• *Problems with Enforcement*: we raise concerns about the Online Safety Bill’s regulatory model of enforcing a Code of Practice. Although we can only speculate about the content of such a Code of Practice, we argue that a Code of Practice is in principle an inapt tool for a dynamic digital ecosystem. Instead, we suggest that an ethical design approach would be better suited to resolving the problems that the Online Safety Bill is meant to address, and provides developers with the flexibility to adopt a plurality of preventative measures.

Our intended readership are those with an interest in the regulation of digital services, both in industry and in policy-making. This is not limited to policymakers: we argue that choices at a technical level (i.e., what tools to use to make the platforms safer) have important moral and political implications, and so it is important that those in data science and machine learning understand the consequences of these tools. We intend to contribute to an increasing debate with the hope of improving the UK’s digital regulation. The arguments we present here reflect our critical opinion, rather than an exposition of scientific fact, and so we invite critical reply to our claims here. We begin with an overview of the legislation. Those interested in our commentary should move directly to the section “Measuring the Bill through proportionality and necessity.”

## An overview of the Online Safety Bill

In 2020, the UK government issued the Online Harms White Paper with the purpose of creating a safer internet ecosystem and repairing public trust in digital platforms and search services. The White Paper has subsequently led to the Online Safety Bill, a draft piece of legislation giving more regulatory substance to the ambitions set out in the original White Paper. In this section, we offer a selected overview of the proposed legislation, where our selection of emphasis is presented with a view to offering commentary on those specific elements.

The Bill’s approach is to place a duty of care on internet service providers of both user-to-user services in which users interact with each other online, such as the manner in which users typically interact on platforms like Facebook and Twitter, and search services that index information and allow users to navigate the internet, such as Google and Bing. The duty of care is framed in broad terms in the Bill, but it is composed of three distinct duties:^7^

1. *To protect users from illegal content* (section 9): although the production and dissemination of CSAM and terrorist propaganda are already illegal, the purpose of the Bill is to place a duty of care upon service providers to control the digital space with a view to limiting the potential spread of illegal content. It is unclear what the Bill is able to accomplish in this space that is not already possible under the existing legal landscape.
2. *To take additional protective measures to make their site safe for children, if their service is likely to be used by children* (section 10). Children on the internet are vulnerable to grooming, cyberbullying, encouragement to self-harm, and harm to their mental health. The purpose of the Bill is to place a duty of care on service providers to ensure the safety of children on their platforms. The litmus test for “a service likely to be used by children” is not well defined.
3. *To take additional measures to protect all users from content that is harmful without being illegal, if the service is of a sufficient reach and magnitude* (section 11). Some online interactions, while legal, can nevertheless be harmful. This includes online campaigns of harassment that are not covered by existing criminal law, and the proliferation of disinformation (fake news). The purpose of the Bill is to place a duty of care upon service providers to ensure that their users are protected not only from unlawful harm, but also from lawful and harmful content.

In the sections below, we highlight key features of the legislation and the duty it places on service providers, with a focus on those features that we argue are problematic. The duty is justified with reference to the role of user-to-user and search services in proliferating harm, and so it is ultimately a duty owed to the users of internet services (albeit arbitrated by the government and regulator). The duty requires services to comply with Codes of Practice, and to report their compliance to the regulator to evidence their execution of their duty. Concomitantly, the regulator, Ofcom, is empowered to enact and enforce the Codes of Practice, although the Minister has the power to direct this enactment.

### Rationale for duty

In its initial White Paper on the subject (then under the auspices of the Online Harms Bill), the government presented both evidence of the extent of online harms and a precis of existing efforts to regulate online harms. We survey these reasons and highlight potential lacunas here by way of exposition before our critical analysis in section 2, which assesses whether the Bill’s rationale as given demonstrates that tighter regulation of service providers is necessary for reducing harm.

The government presents the following online harms as the crucial impetus for the Bill:

• *Child sexual exploitation and abuse online*: the White Paper cites the Internet Watch Foundation’s statistics regarding the circulation of CSAM. The White Paper notes that, of the 80,319 cases of CSAM confirmed by the IWF, 43% of the children involved were between 11 and 15, 57% were under 10, and 2% were under 2.^2^
• *Terrorist content online*: the government’s concern is that the internet allows the proliferation of terrorist propaganda. The White Paper repeats the Rt Hon Amber Rudd’s claim that five of the terrorist incidents in the UK in 2017 included internet elements, implying that they were radicalized by international groups, such as ISIS and Daesh.^3^
• *Content illegally uploaded from prisons*: the White Paper claims (without citation) that there is an increase in the amount of content transmitted illegally from prisons.
• *The sale of opioids online*: the White Paper cites the National Crime Agency’s statistics that there have been at least 146 opioid-related deaths in the UK since 2016. It claims (without reference) that opioids are sold on “several well-known social media sites.”
• *Cyberbullying*: the White Paper cites National Health Service data that one in five children aged 11–19 has experienced cyberbullying. Of those who experienced cyber-bullying, the White Paper claims that the propensities of victims towards social anxiety, depression, suicidality, and self-harm were all higher than corresponding cases of ordinary bullying.
• *Self-harm and suicide*: the White Paper cites academic research that showed that 22.5% of young adults reported suicide and self-harm-related internet use; 70% of young adults with suicidal intent reported related internet use; approximately a quarter of children who presented to hospitals following self-harm, and of those who died of suicide, reported suicide-related internet use.^8^
• *Underage sharing of sexual imagery*: the White Paper cites statistical evidence that suggests that between 26% and 38% of teenagers have sent sexual images to partners, whereas 12% to 49% of teenagers have received sexual images from partners.
• *Online disinformation*: the White Paper cites a Reuters report showing that 61% of people want the government to do more to distinguish between real and fake news. The White Paper does not provide statistical evidence as to the prevalence of disinformation, but cites the Russian state’s use of disinformation as an example.
• *Online manipulation*: the government’s concern is that the introduction of artificial intelligence will increase the prevalence and effectiveness of psychological manipulation. The government cites the need to replicate the regulation of manipulation in other media, such as the Broadcasting Act 1990, which prohibited subliminal advertising.
• *Online abuse of public figures*: the government cites the disproportionate amount of abuse received by female public figures. It cites an international survey that found that two in every three female journalists were harassed online, including receiving death threats, cyberstalking, and obscene messages.

Where these activities are already illegal (as in the case of CSAM or terrorist recruitment and propaganda), *the purpose of the legislation is not to prohibit the act itself but to establish the duties of online services to protect users from illegal content.* This deviates from the existing regime, established by the EU’s e-Commerce Directive, which exempts services from liability for illegal content, unless they know about the unlawfulness of the content or have sufficient information to conclude that it is unlawful and do not act expeditiously to remove the content. We cover the form of this duty in the next two subsections.

Where content is legal, the White Paper argues that it falls in a regulatory lacuna, since legal (but harmful) content is already subject to statutory regulation (typically overseen by Ofcom) when it is presented on other platforms, including live television, catch-up television, and subscription services. The Bill, therefore, fills the regulatory gap by extending similar regulatory control over the dissemination of content on user-to-user services.

### Scope of the duty

The scope of illegal content is, of course, specified already by preceding legislation, and includes material that promotes terrorism or that is classified as CSAM. However, the Online Safety Bill adds a new layer of liability to these activities because it requires that services adhere to an additional layer of compliance measures and empowers the government to hold named directors accountable for their services’ failures to comply.

*Ordinary sensibilities*: what is more novel is the Bill’s focus on harms that are not illegal. Citing online harassment in its White Paper, the government has suggested that there is a need to regulate behavior that is not prohibited or regulated otherwise, including speech that does not fall within the remit of hate speech. The Bill requires that service providers not only protect their users from illegal content but also from this species of content that would otherwise be legal, as long as it can reasonably be construed as potentially harmful to children or adults of “ordinary sensibilities.”

The scope of this content is potentially very wide. According to the Bill, this duty should be triggered if:

the provider of the service has reasonable grounds to believe that the nature of the content is such that there is a material risk of the content having, or indirectly having, a significant adverse physical or psychological impact on an adult of ordinary sensibilities.

The Bill, therefore, uses the “reasonableness” standard that pervades English common law. This standard sets a variable threshold that focuses on the perspective of an epistemically responsible agent. This definition is further supplemented by the following stipulation in the Bill:

[A] risk of content “indirectly” having a significant adverse physical or psychological impact on an adult is a reference to a risk of either of the following—(a) content causing an individual to do or say things to a targeted adult that would have a significant adverse physical or psychological impact on such an adult; (b) content causing an adult to act in a way that—(i) has a significant adverse physical or psychological impact on that adult, or (ii) increases the likelihood of such an impact on that adult.

This framing is noteworthy both because it includes in its scope a responsibility on the part of services for content that is not illegal otherwise, but also because it provides such wide interpretive scope depending on how one chooses to construe “reasonableness.” We return to this issue later in this paper.

### Enforcing the duty

The Bill and White Paper assert that service providers have, in principle, a duty of care to protect their users; to discharge this duty, the Bill stipulates that services must abide by Codes of Practice. *The Bill itself does not specify the content of the Codes of Practice. Instead, it delegates this authority to the industry regulator, the Office of Communications (Ofcom).* Ofcom is an independent industry regulator, although its chair is politically appointed.

While Ofcom has the power to set the Codes of Practice, the Bill vests the Minister of State with the power to veto the Codes of Practice, or to order Ofcom to modify the codes so that they align with “government policy.” The Bill, in effect, grants the Minister of State wide powers to direct the Codes of Practice.

Parliament, by contrast, has relatively little oversight over the Codes of Practice. The Bill adopts a negative form of parliamentary oversight with regard to the Codes of Practice: Parliament is assumed to have consented to the Codes of Practice unless its members propose and pass a vote to reject the Codes. Scrutiny of the Codes of Practice, therefore, is the exception, rather than the default position of Parliament.

### Assessing the Bill

In this section, we have surveyed the government’s own rationale for the Bill, as well as assessing the scope and content of the duties of care that the Bill imposes. Our focus for the remainder of this paper will be on:

(1) the causal claims in the government’s rationale for the Bill and whether they justify the necessity of the legislation;
(2) the authority that the Bill vests in the Minister and in Ofcom;
(3) the wide scope of content that is included in service providers’ duty of care per the Bill; and
(4) the Bill’s reliance on Codes of Practice to be prescribed by Ofcom in conjunction with the Minister.

In the sections that follow, we argue that: the government’s rationale for the Bill provides insufficient justification for the necessity of this intervention, and that more consultation and justification are needed; the Bill grants far-reaching powers of interference to the executive; the scope of content covered by the Bill is worryingly broad; the emphasis on Codes of Practice is inapt; and that the Bill creates potential obstacles for small and medium enterprises.

## International comparisons

It is worth comparing the Bill with its international equivalents, since legislators in a number of jurisdictions have sought to regulate content-moderation on social media platforms. These proposed legislative interventions provide us with a useful set of benchmarks against which to measure the Safety Bill.

The Parliament of the European Union is currently considering the proposed Digital Services Act (DSA) to address content moderation in the EU.^9^ Like the OSB, the DSA is aimed at protecting the human rights of citizens of the EU online. However, the proposal differs in several important regards. The DSA stipulates more detailed, design-based duties with regard to legal but harmful content: user-to-user services must contain clear and accessible terms and conditions, content-reporting procedures, and appeal procedures following content or user removal, as well as requiring large platforms to cooperate with “trusted flaggers” (drawn from expert and professional institutions) who report harmful content (section 3). The DSA also requires very large platforms to perform assessments of their systemic risks, including systemic design features that threaten the exercise of fundamental rights, to declare the parameters of their recommender systems, and to evidence their mitigation strategies for minimizing system risk (section 4). Therefore, with regard to legal but harmful content, the DSA is concerned only with systemic design features of user-to-user services.

To help enterprises, the DSA empowers the European Commission to issue guidelines for the fulfilment of their duties stipulated in the Act (article 27). This differs from the Codes of Conduct in three important respects. First, the Commission is obliged to compose the guidelines in collaboration with the services affected and civil society organizations representing stakeholders. Second, the guidelines are meant to represent best practice in the industry, but enterprises can deviate from the guidelines with sufficient reason. Third, the guidelines do not create new duties: rather, they are meant only to help enterprises easily navigate the duties already established in the Act.

The DSA takes a comparable approach to the Platform Accountability and Consumer Transparency Act (PACT Act), which has been proposed in the Senate of the United States.^10^ Concerning legal but harmful content, The PACT Act, like the DSA, focuses on design features of user-to-user services: the Act stipulates transparency and process requirements for acceptable use, complaints, and content moderation, requiring services to submit transparency reports (section 5).

By contrast, the national legislatures in Brazil^11^ and India^12^ have both considered much stricter regulation of content monitoring online. The Brazilian executive issued a Provisional Measure 1068 to restrict content removal by social media platforms, limiting removal only to cases of nudity, violence, narcotics, and incitement to crime, thereby preventing social media platforms from removing disinformation (such as President Jair Bolsonaro’s COVID-19 disinformation removed by Facebook, Twitter, and YouTube).^13^ The Indian government has similarly issued a number of regulations, including the Information Technology Act^14^ and Information Technology (Intermediary Guidelines and Digital Media Ethics Code) Rules of 2021,^12^ which direct user-to-user services to remove a wide range of content, including material that threatens the sovereignty of the Indian state, to use algorithmic systems to monitor and remove harmful content, and to trace encrypted messages to limit online anonymity. Activist groups have claimed that these measures are aimed at curbing dissent against the government, resulting in what they call “digital authoritarianism.”^15^

These comparators are useful in framing the different degrees to which governments have chosen to interfere with services’ content monitoring and moderation. The US and EU models are focused on design choices that empower users by making the terms and procedures of user-to-user services transparent and accessible. The Indian and Brazilian models, by contrast, are focused much more explicitly on directing the content that is permissible on user-to-user services. The UK government has intimated its inclination toward the former approach, but this remains relatively underdeveloped in the Bill itself, as we discuss in the following sections.

## Measuring the Bill through proportionality and necessity

The Online Safety Bill will necessarily limit individuals’ right to freedom of expression, and place costly positive duties on entrepreneurs that limit their free enjoyment of their property (not to mention downstream effects on their competitiveness). These rights are enshrined in the European Convention on Human Rights (article 10) and the subsequent Paris Protocol (article 1). However, we are concerned here not with the legal right—particularly since the UK parliament reserves the right to enact legislation that is incompatible with its commitment to the Convention. Rather, we are concerned with the normative right that underpins the aforementioned legal creations. As our point of departure, we assume that individuals are vested with natural rights to free expression and use of their property.

These rights, of course, are not absolute or insurmountable: it is, by way of exception, permissible to infringe upon these rights in the presence of sufficient countervailing justification. Rights provide “moral breakwaters”^16^ that protect individual and collective interests, but the breakwaters can always be overcome with sufficient justification. However, since rights function as breakwaters, they cannot be limited anytime that infringement causes a net good. Rather, rights are only defeasible when the costs of respecting the rights is significantly greater than the cost of transgression.^17^ In other words, rights establish a default position, and deviation from this default requires significant and extraordinary reason. This sets the standard of evidence that the government should (normatively, albeit not legally) adduce to support the infringement of others’ rights: their intervention should not simply produce a net good; rather, the net good must be sufficiently weighty to offset the cost of intentional rights infringement.

In normative theory, rights infringements are subject to the requirements of necessity and proportionality.^18^ Necessity permits rights infringements only as a last resort: rights cannot be transgressed if there are less harmful means available to achieve the same end. Proportionality permits rights infringement only if the expected outcome of the infringement is commensurate with the cost of the infringement—in other words, there must be an apt “fit” between means and ends.

What we want to suggest here is that the evidence presented by the government in justifying the Online Safety Bill (and the infringements it entails) does not meet the thresholds of necessity and proportionality. Given the stringency of the rights affected, it is critical that the government should adduce clear and convincing evidence that its proposal satisfies these requirements. To this end, we suggest that the government’s rationale for the Bill raises several general concerns that cast doubt upon the proportionality and necessity of this intervention. We list these concerns below.

### Proportionality issues

In several instances, the White Paper cites genuine concerns about the contributions of user-to-user and search services. However, if we investigate these claims more closely, there is a mismatch between the putative justification of the White Paper and the remit of the legislation. In brief, the problem can be framed as follows:

• the interventions in the Bill do relatively little to resolve the concerns raised by the White Paper;
• to resolve the concerns thoroughly would require interfering with legitimate internet use.

This is a problem of proportionality, because the expected benefit of the intervention does not fit the magnitude of the rights infringement entailed by the intervention.

Consider, first, the problem of bullying. The government is right to want to address the problem of cyberbullying: one in five children report being subject to cyberbullying.^19^ However, the Bill only addresses a fractional part of this problem, because 90% of cyberbullying occurs in private messages between schoolmates.^19^ The Bill, as it is, is unlikely to resolve the problem that the White Paper outlines, since private communications fall outside of the scope of the Bill. Of course, the government could resolve the problem more effectively by expanding the remit of the Bill to include private communications, but this would clearly constitute an unacceptable breach of individual rights, and would require services to break the encryption of private messaging, which would have extremely deleterious privacy costs.

A similar problem plagues the government’s ambitions concerning suicidality and self-harm. The White Paper is correct in emphasizing the correlation between internet use (particularly of search and user-to-user services) and suicidality, depression, and self-harm. However, it is again unclear whether the Bill’s interventions present an effective and proportionate solution to the problem. First, although internet use is strongly correlated with suicidality and depression, this correlation is predominantly due to the effects of sleep loss and private cyberbullying, rather than exposure to suicide-related content (which has a very low correlation with suicidality).^20^,^21^ Second, where individuals experiencing suicidality have accessed suicide-related content prior to self-harming, the preponderance of this content has not been user-to-user content, but rather fact-based websites.^20^ It is unclear, therefore, whether the Bill as it is will be able to resolve much of the problem of suicidality and self-harm. Again, the government would have to make much further-reaching interventions to get to the real causes of the problem, but this again would be at the cost of interfering with individual liberties.

### Necessity issues

The second point of concern is that, even where the Bill’s interventions are effective in preventing or mitigating harm, there are interventions available to the government that would interfere less with individual rights. This would render the interventions in the Bill unnecessary, since they do not constitute the least costly means of addressing the intended harm.

For the purposes of measuring necessity, it is useful here to benchmark the interventions in the Bill to similar interventions in comparable legislation, such as the DSA. The DSA does not focus on content monitoring or moderation by platforms themselves, but rather focuses on setting out clear and easily accessible mechanisms for users to register complaints and to flag content that contravenes the terms and conditions of the service (sections 3 and 4). This approach causes less interference with the rights of individual users, because it means that they are not monitored by default, as well as imposing less burdensome duties on service providers, since they do not have to develop monitoring mechanisms (which we cover later in this paper). This approach also reduces interference by diminishing the possibility of removing non-harmful content, since only content flagged by users (rather than by monitoring AI) will be picked up.

The DSA’s approach also avoids the Bill’s more costly solution of de-anonymizing user-to-user services. The Bill proposes limiting the ability of individuals to use user-to-user services anonymously as a means of harm prevention. While the restriction of the ability of individuals to be anonymous might have benefits in terms of holding individuals more accountable for their actions, which could promote less toxic interactions online, the limiting of anonymity might be damaging to those who rely on the lack of identification online to access support.^22^ Chat rooms and support groups can be beneficial for those experiencing mental health issues and one of the features that can increase their effectiveness is the ability to be anonymous. Individuals are able to access support without their identity being revealed, meaning they are free to discuss their experiences without being concerned about their employer or family finding out, for example. It is unclear how the Bill would balance minimizing harm facilitated by anonymity while avoiding bringing harm to those who rely on anonymity to access support. Relying instead on user reporting is a means of empowering individuals against online harm without limiting their ability to interact freely and anonymously online when it is beneficial to their wellbeing.

## Democratic deficit

Our second concern relates to the enforcement of the duties set out in the Bill. We argue that the structure of the Bill grants undue power to the executive, and deprives the public of the opportunity to exercise democratic oversight of the Bill’s content.

In designing the structure of its regulation, the Bill assigns to Ofcom the power to issue Codes of Practice that will determine how user-to-user and search services are to fulfil the more abstract duties set out in the legislation. As mentioned in section 1.3, the Bill also grants the Minister the power to interfere with the Codes of Practice by exercising a veto power or by directing Ofcom to align the Codes of Practice with government policy.

Given that the government will be able to punish services and individuals who are derelict in their duties, and will inform what information can be shared and received on the internet, the Codes of Practice have significant implications for the rights of internet users and services. The Codes of Practice can make information more or less difficult to communicate. Our concern is that the stringency of these restrictions is dependent upon the regulator and ultimately the Minister, with little oversight from Parliament or the public (although the Department has suggested it will consult stakeholders). Since the Minister can direct the Codes of Practice, they are effectively granted the authority to determine how user-to-user services control speech on their platforms.

Our concern is that this creates the possibility of a democratic deficit in the Bill: the Minister retains sweeping powers to interfere with the limits and regulation of speech on the internet’s key platforms, with Parliament playing only a minimal negative oversight role. This power is sweeping since the remit of the Bill is wide (particularly in defining the “harmful but legal” content for which services are responsible). This means that the Minister has significant power to interfere with an important set of rights (including free speech and free press) without the particulars of their interventions being vetted by Parliament or subject to public scrutiny. We suggest that this amount of power is susceptible to abuse, and does not accord with the Bill’s vision of a free internet.

## New powers and duties

The Bill empowers Ofcom to enforce both services’ duties concerning illegal content, as well as their duties concerning “legal but harmful” content. We have particular concerns about the duty placed upon services to control “legal but harmful” content, given the breadth of the definition of what counts as harmful. Here, we argue that the breadth of the content covered by the Bill would be better addressed by ethics-by-design procedural mechanisms, rather than content-specific regulation.

The Bill defines harmful content in Part 2, Chapter 6, Section 46, repeating its formulation in defining content that is harmful to children. The formulation in this section has a few noteworthy features. The first is that it includes content that causes “indirect” harm and extends the remit of harm to psychological (and not just physical) harm. The second, more worrying, feature of the formulation is that it defines harm in terms of the reasonable understanding of a person of “ordinary sensibilities.” The introduction of the reasonableness element imports an interpretive element into the duty without inserting clear boundaries delineating the scope of the duty, which—as we explain in this section—is troublesome for a top-down approach to regulating harm.

Our concern here is not with the cogency of a wider definition of harmful speech as such. It is plausible, we think, to extend the remit of “harmful speech” beyond the remit of what counts in law as “hate speech.” However, in our opinion, the Online Safety Bill does not specify this remit with sufficient clarity for the purpose for which it is deployed. This formulation has the capacity to include a vast sweep of material that would be undesirable to limit. Whether an individual piece of content counts as “harmful” on this definition will depend significantly on the context of its use. It is this matter of interpretation that, we think, opens the possibility of over-censorship when enforced by a top-down approach in which the government specifies a list of harmful content or algorithmic systems are used to detect harmful content (as in the Brazilian and Indian cases).

Consider two examples that pose particular interpretive difficulty: manipulation and disinformation:

• *Manipulation*: “nudging” refers to features of choice architecture that alter an individual’s behavior in a predictable way^23^ and, although disputed, can be said to be a form of manipulation of the choices people make.^24^ Nudging can take simple forms, including the placement of products in the supermarket, with branded products being placed at eye level to encourage consumers to spend more.^25^ Nudging has also been used in public health interventions, including in reducing meat consumption^26^ and the encouragement of healthier food purchasing,^27^ as well as in pro-environmental behavior.^28^ Evidently, nudging (and manipulation) is present in analog settings and is not novel to online, algorithmic-driven settings, such as social media. Indeed, traditional information flow theory can be adapted to algorithmic nudging.^29^ This is not to say that more analog forms of manipulation and algorithmic manipulation, or algorithmic personalization as it is commonly referred to in the literature, are exactly equivalent. Algorithmic manipulation can be more covert than human manipulation,^29^ which, in part, is due to the often black box nature of algorithms.^30^ It also allows a greater level of personalization since the large number of data points collected about an individual from their online activity enables highly accurate profiles to be generated about them.^31^ However, again targeted advertising, which falls within the remit of nudging/manipulation,^32^ can occur offline, although with less personalization; television advertisements are targeted at the intended audience of the station, including targeting to children,^33^^,^^34^ and the placement of billboards enables targeting to specific demographics.^35^^,^^36^ It is not clear, in the context of the Bill, how we are to distinguish meaningfully between legitimate techniques of persuasion and bad faith manipulation.
• *Disinformation*: the White Paper provides seemingly contradictory directives with regard to limiting disinformation. The White Paper claims, simultaneously, that user-to-user services have an obligation to “improve how their users understand” the “trustworthiness” of news (7.29), but it also confirms that the purpose of regulation should be “protecting users from harm, not judging what is true or not” (7.31). It is not clear how these two imperatives can be squared. If “harm” is simply content that is already illegal (including hate speech, defamation, unlawful political interference), then it is unclear what additional protection the Bill will contribute. However, if “harmful” is construed more widely, then the Bill will invariably have to set parameters for the kind of information that counts as disinformation (rather than simply misinformation). This is a delicate interpretive task that depends upon the context in which information is disseminated, and so requiring stricter monitoring will require tradeoffs in which services will have to limit expression.

Our concern here is that the top-down monitoring of content (either by the government or by AI deployed by services) —given these interpretive difficulties—will increase the risk of excessive censorship. Whether an individual piece of content constitutes “harm” by the definition above will be highly sensitive to the context of its use. The context sensitivity of this definition suggests important technical difficulties for enterprises. Enterprises will presumably have to develop tools to scan content for a number of harms. Automatic detection of harm is an open problem far from being solved.^37^ The academic community researching automated detection of cyberbullying, for example, has made appeals for more universal and specific criteria concerning cyberbullying definitions, more consistent evaluation methods, and better-quality datasets.^37^^,^^38^ It is easy to see how larger companies with access to more data and highly skilled technical personnel would be better placed to solve the task, whereas smaller firms will struggle to meet this serious technical task.

Given the importance of the issue for the safety and human rights of users, we endorse the research community’s call for a clearer set of criteria for “harm.” We also recommend supporting the creation of universal tools, which could be achieved by collecting or sharing datasets and existing technologies and would remove the burden from small and medium enterprises. However, more generally, it is our opinion that an ethics-by-design approach would mitigate much of this concern, because it would empower users to inform the moderation themselves with the help of the appropriate procedural mechanisms.

## Codes of Practice

Our final concern relates to the Bill’s focus on Codes of Practice deferred to Ofcom as the main regulatory mechanism. Our concern here is that the legislation’s open-ended references to Codes of Practice opens the possibility of inappropriate regulatory tools. As we intimate in the previous section, our concern here is that the Codes of Practice leave open the possibility that regulation will restrict particular pieces or kinds of content. This would, of course, place an unduly onerous burden on service providers, and hold them responsible for activities on their sites for which they should not be held liable.

We note that the GDPR and the DSA include a similar mechanism that permits regulators to establish Codes of Conduct or best-practice guidelines.^39^ However, it is important to note that the Codes in these cases do not establish new rules that are not grounded in the legislation: rather, it provides efficient means for enterprises to comply with their duties established by complex legislation. Our concern is that the Online Safety Bill is sufficiently open-ended that the Codes of Practice will, in this case, amount to the creation of new rules, since the duties in the Bill are multiply realizable and open to a wide range of interpretations. This is because the Bill outlines only in broad terms the duties that services have to protect users, but does not prescribe (as the DSA and GDPR do) which features of their platforms are in the scope of the regulation (i.e., whether they have a duty to monitor and moderate specific pieces of content, or whether they only have a duty to adjust the design features of their services).

The most sensible approach, we argue, would be to adopt an ethical design approach that (1) focuses on the ethical features of the design process and (2) provides services with sufficient space to adopt flexible and innovative solutions to the social problems present on their platforms. A Code of Practice runs the risk of focusing less on design, and rigidifying the solutions that providers can use to solve problems.

In a recent memorandum on the topic, the Department of Digital, Culture, Media, and Sport has indicated that they will focus their Codes of Practice on design and process features of user-to-user and search services.^40^ However, we would appeal to Ofcom, Parliament, and the DCMS to concretize this commitment to assuage concerns about content-specific censorship. It is important for the purposes of clarity that this be confirmed.

Compared with those jurisdictions that have taken a content-specific approach to regulation, the Online Safety Bill is less stringent and specific. Indeed, India’s Information Technology Rules,^12^ which echo the sentiment of the Online Safety Bill, defines content that must be reviewed under the rules, listing discrimination, psychotropic substances and smoking, imitable behavior, such as content depicting self harm and offensive language (such as expletives, nudity, sexual content, and violence). The Rules also require the appointment of a Chief Compliance Officer in social media companies to ensure compliance and cooperation, and identification of the first poster of the unacceptable content in some cases. Likewise, the Russian law *On Information, Information Technologies and Information Protection* requires social media sites to monitor and restrict content related to material concerning the advertising of alcohol and online casinos, disrespect for society, information on drug synthesis and production, and suicide. Violations are required to be registered with the Federal Roskomnadzor register.^41^

## Concluding remarks

We share the government’s concerns about the potential hazards of the internet, particularly with regard to vulnerable groups, particularly children. However, this is not the only imperative at stake: it is also important that the government foster an open internet, on which free speech and innovation can flourish. We accept that the state cannot fully satisfy all of these imperatives simultaneously: the state will necessarily have to make tradeoffs between safety, liberty, and innovation.

Insofar as we have been critical of the Online Safety Bill, it has been because we think it has not yet achieved an optimal balance between these imperatives. First, we argue that the Department must do more to justify this legislative intervention: there is a paucity of justificatory evidence for the scope of the Bill in the current White Papers issued in its support. Second, we have argued that the mechanisms of the Bill do not do enough to protect the liberties of platforms and their users, because it effectively defers much of the power to regulate platforms to the Minister. Third, we argue that the Bill imposes overly wide duties on platforms that can be deleterious to smaller enterprises and increase government intervention. Fourth, we argue that it is imperative for the government to commit to an ethical-by-design approach to the duty of care.

It is our opinion that it is possible for the government to correct the problems with the Bill and the White Papers we suggest here without having to make significant sacrifices to its strategic aims. We suggest that these changes—while seemingly small—will have a significant effect on making the internet freer, more open, and more innovative—as well as making it safe.
